# A systematic review of qualitative literature on antimicrobial stewardship in Sub-Saharan Africa

**DOI:** 10.1186/s41256-021-00216-0

**Published:** 2021-08-20

**Authors:** George James Porter, Stephen Owens, Matthew Breckons

**Affiliations:** 1 Faculty of Medical Sciences, Newcastle University, Framlington Place, Newcastle Upon Tyne, UK; 2 Department of Paediatric Immunology and Infectious Diseases, Great North Children’s Hospital, Newcastle Upon Tyne Hospitals NHS Foundation Trust, Newcastle upon Tyne, UK; 3 Population Health Sciences Institute, Newcastle University, Framlington Place, Newcastle Upon Tyne, UK

**Keywords:** Sub-Saharan Africa, Antibiotic resistance, Antibiotic stewardship, Public health, Global health, Qualitative synthesis

## Abstract

**Background:**

Antibiotic resistance is a major problem in every region of the globe and Sub-Saharan Africa (SSA) is no exception. Several systematic reviews have addressed the prevalence of resistant organisms but few have examined the underlying causes in this region. This systematic review of qualitative literature aims to highlight barriers and facilitators to antimicrobial stewardship in SSA.

**Methods:**

A literature search of Embase and MEDLINE(R) was carried out. Studies were included if they were in English, conducted in SSA, and reported qualitative data on the barriers and facilitators of antimicrobial stewardship or on attitudes towards resistance promoting behaviours. Studies were screened with a simple critical appraisal tool. Secondary constructs were extracted and coded into concepts, which were then reviewed and grouped into themes in light of the complete dataset.

**Results:**

The literature search yielded 169 results, of which 14 studies from 11 countries were included in the final analysis. No studies were excluded as a result of the critical appraisal. Eight concepts emerged from initial coding, which were consolidated into five major themes: ineffective regulation, health system factors, clinical governance, patient factors and lack of resources. The ineffective regulation theme highlighted the balance between tightening drugstore regulation, reducing over-the-counter sale of antibiotics, and maintaining access to medicines for rural communities. Meanwhile, health system factors explored the tension between antimicrobial stewardship and the need of pharmacy workers to maintain profitable businesses. Additionally, a lack of resources, actions by patients and the day-to-day challenges of providing healthcare were shown to directly impede antimicrobial stewardship and exacerbate other factors which promote resistance.

**Conclusion:**

Antibiotic resistance in SSA is a multi-faceted issue and while limited resources contribute to the problem they should be viewed in the context of other factors. We identify several contextual factors that affect resistance and stewardship that should be considered by policy makers when planning interventions. This literature base is also incomplete, with only 11 nations accounted for and many studies being confined to regions within countries, so more research is needed. Specifically, further studies on implementing stewardship interventions, successful or not, would be beneficial to inform future efforts.

## Background

According to The World Health Organisation (WHO), Antimicrobial resistance (AMR) directly threatens frontline clinical care, limiting our ability to treat infections as well as increasing the risks of interventions such as surgery and chemotherapy [1]. AMR also limits development by draining the global economy and reducing productivity due to sickness [1]. While considerable research is dedicated to the epidemiology of resistant organisms and novel therapeutics, another important facet is the clinical and behavioural factors driving resistance [2, 3].

AMR is a growing problem in Sub-Saharan Africa (SSA) and is complicated by a lack of data [4]. One systematic review analysing resistance prevalence in Africa found that there was no data for 40% of African countries [4]. This is partly due to a paucity in quality-assured microbiology laboratories in the region, along with AMR being a low priority compared to other public health concerns [5, 6]. Furthermore, according to Essack et al. [7], only 4.3% of countries in the WHO Africa region have national AMR plans while 14.9% have national infection prevention and control policies.

The data that are available demonstrates a significant problem. One systematic review found that *E. coli* isolates had a median resistance of 88.1% to amoxicillin and 80.7% to trimethoprim, while 34% of *H. influenzae* isolates were resistant to amoxicillin [4]. There is also considerable resistance to WHO-recommended first-line drugs [8]. The WHO-recommended treatment for sepsis in children under 2 months of age is ampicillin and gentamicin [8]. According to systematic review data, the median non-susceptibility rate of *Klebsiella* isolates from paediatric infections in SSA was 100% (IQR 71–100) for ampicillin and 49% (IQR 48–58) for gentamicin [8]. Additionally, the WHO acknowledge that in many developing countries illnesses such as pneumonia and dysentery can no longer be treated with first-line medications [1]. Without prompt action these trends will likely worsen and countries with stretched health resources, whose patients cannot afford the required second or third-line antibiotics, will be disproportionately affected.

There is considerable research dedicated to combatting AMR, especially in resource-limited settings [1, 5]. The behaviours which drive resistance are thus relatively well defined [5]. Within SSA there are many examples of cross-sectional surveys of the prevalence of these behaviours, which include patient self-medication, over-the-counter (OTC) sales of prescription-only antibiotics and over-prescribing of antibiotics [9, 10]. While these surveys identify what behaviours cause resistance it is also important to identify the underlying drivers of these behaviours. A qualitative approach can provide rich data from patients, healthcare staff and public health professionals describing why resistance-promoting behaviours happen. These data are of value to policy-makers; highlighting key determinants and context of antibiotic resistance.

Systematic review and synthesis of qualitative data is a reasonably new methodology but one that has gained acceptance in scientific literature. Indeed, the Cochrane collaboration recently called reviews of qualitative evidence a “new milestone for Cochrane” [11]. There are many methods of qualitative synthesis, each having evolved from different fields [12, 13]. There is little consensus on the best method, with each having their own strengths and weaknesses [12]. Studies must therefore be designed based on the questions they intend to answer [12, 13].

There have been significant efforts to research barriers and facilitators to antimicrobial stewardship (AMS) in Sub-Saharan Africa, but to our knowledge no synthesis of qualitative literature has yet been published on the subject. The objective of this review is to highlight barriers and facilitators to antimicrobial stewardship and sociocultural factors driving antimicrobial resistance-promoting behaviour in patients and healthcare staff in Sub-Saharan Africa. We hope that this will provide policymakers with a more comprehensive view of the underlying factors which need to be addressed to curb AMR in this region and highlight gaps in the literature.

## Methods

### Research methodology

The methodology for this review was guided by the Preferred Reporting Items for Systematic Reviews and Meta-analyses (PRISMA) guidelines and checklist [14]. Selection of methodology was guided by the review written by Bearman and Dawson [13]. Specific information on how to extract, code and analyse qualitative themes was sourced from Butler et al. and Seers [15, 16]. Given that we are attempting to summarise current literature and identify key recurrent messages, thematic analysis was selected as our method of qualitative synthesis [13].

For the purposes of this review, the United Nations Development Programme’s definition of Sub Saharan Africa was used to define geographical inclusion [17].

### Search strategy and selection criteria

Ovid online was used to search Embase and Ovid MEDLINE(R). There were no restrictions with respect to date of publication. Results were limited to publications in English. The last search occurred on 19/05/2020. Multiple searches were conducted, used terms included ‘Antibiotic Resistance’ or ‘Antimicrobial Stewardship’ along with “Africa South of the Sahara” and ‘Qualitative Research’. All terms were exploded and then combined with the Boolean operator AND. Medical subject headings (MeSH) terms were also included. The full search strategy for Embase and MEDLINE(R) can be found in “Appendices 1 and 2”, respectively.

The authors also searched for cross-sectional surveys, as some of these studies had qualitative elements to them. This was done systematically via similar keywords to above but substituting ‘qualitative research’ for ‘cross-sectional survey’. The references of included studies were also searched for additional papers. Studies first underwent abstract screening to ensure they met the inclusion criteria and then full-text screening and data extraction (Table 1).

**Table 1:** The inclusion criteria used in the original literature search and screening process and their explanations

Inclusion criteria	Explanation
1. Must be a qualitative study	This includes in-depth interviews, focus groups, ethnography and other qualitative techniques It does not include cross-sectional surveys, surveillance data or clinical trials unless they contain a qualitative component as listed above
2. Must pertain, at least in part, to data gathered in Sub-Saharan Africa	This allows multi-country studies, in which case the secondary constructs from SSA nations only were extracted SSA was defined as per the United Nations Development Programme [13]
3. Must be directly relating to causes of resistance-promoting behaviours	This includes barriers/facilitators to implementation of stewardship schemes and social and cultural determinants of resistance-promoting behaviours It does not include discussions exclusive to HIV or Malaria, as these were seen as separate issues with different socio-cultural context
4. Reporting primary research findings	This excludes review articles

### Critical appraisal

Critical appraisal was conducted by GJP. All included studies were evaluated using the CASP (Critical Appraisal Skills Programme) qualitative research appraisal tool, a 10-item checklist covering domains including research design, data collection and analysis [18]. The first 9 are answered ‘yes’, ‘no’ or ‘can’t tell’ and the remaining question asks for a subjective evaluation of the value of the study [18]. Studies were scored principally by the first author. The first three CASP questions provide a screening tool to evaluate if the research question of the study can or should be assessed via qualitative methodology. Failure on this section would result in exclusion of the study. Meanwhile, the latter questions were the taken into account when resolving disagreements between studies.

### Data extraction and synthesis

Data were defined as secondary constructs, that is to say the researcher’s interpretations and conclusions, rather than direct quotes from study participants. Thematic analysis, as described by Seers and Bearman and Dawson, was then conducted [13, 16]. The first author read each paper and coded secondary constructs, grouping them into various concepts. These concepts were then reviewed and simplified into themes once all studies had been coded. The final models were evaluated by senior authors (SO and MB) to ensure they were consistent with the source material.

The summary measure of this review was refutational and reciprocal synthesis across studies about the barriers to AMS implementation and the causes of resistance-promoting behaviour. Other information collected included country of origin, the number and occupation of interviewees in the study and information about healthcare staff and patient’s perceptions of AMR as a threat (Table 1).

## Results

### Included studies

**Fig. 1:**
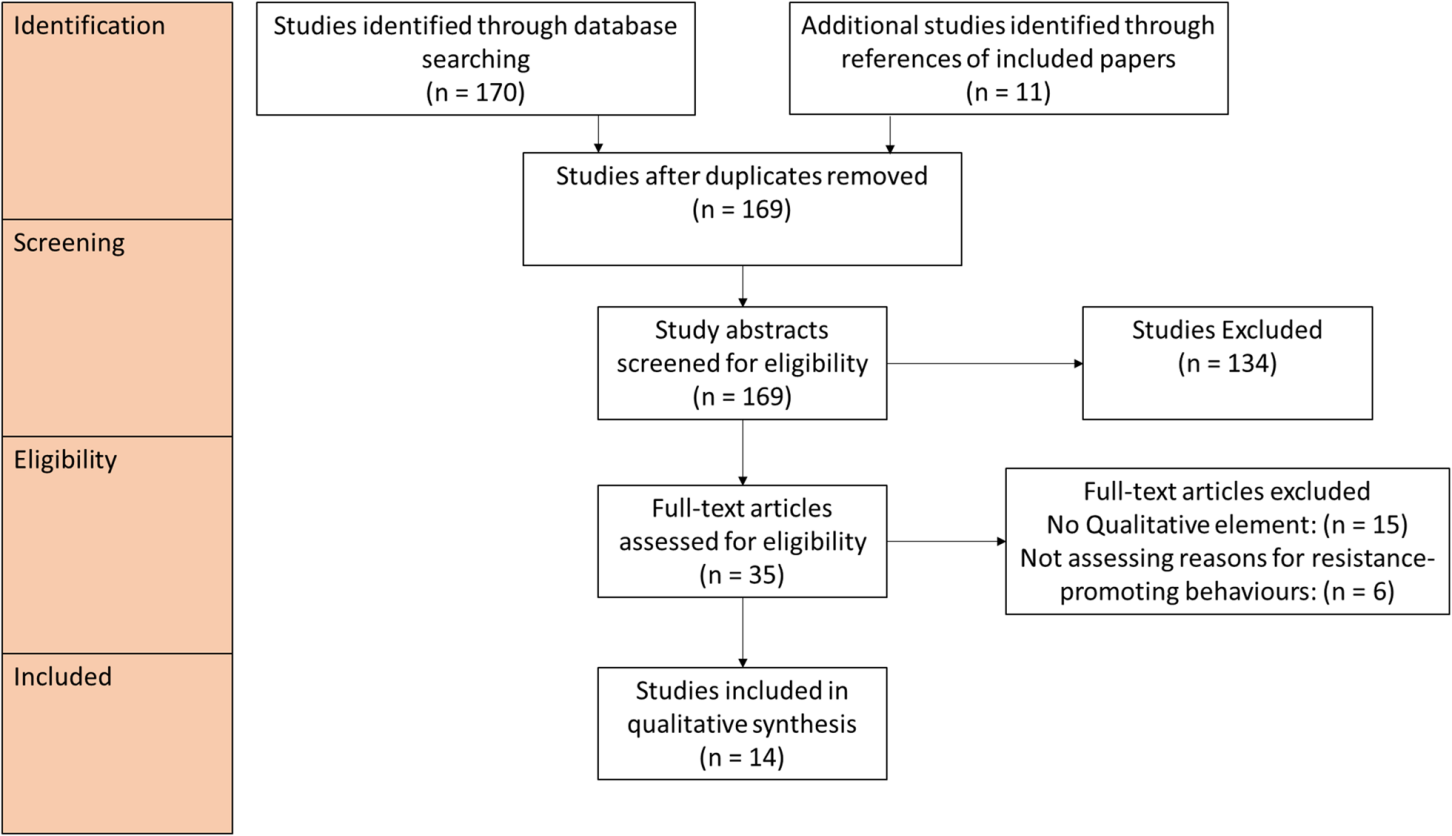
PRISMA flow diagram illustrating the screening process for studies found in the literature search

Excluding duplicates, the literature search yielded 169 results, of which 138 were excluded on abstract screening. This resulted in 35 papers which underwent full-text review and 14 papers included in final analysis. A PRSIMA flow diagram can be found in Fig. 1. Data were found relating to 11 of 46 SSA countries.

The average CASP score was 7.8/9 and the lowest score was 7/9. The most common omission was a lack of discussion of the relationship between researchers and participants, which occurred in 5 of the 11 studies. Additionally, 4 studies either did not explicitly detail recruitment strategy or used either subjective or selective recruitment criteria. Table 2 illustrates the results of the critical appraisal.

**Table 2:** Table illustrating the results of the critical appraisal process including manuscript information, the number of and occupation of participants and their critical appraisal score, including justification for points lost

Manuscript			Interviews		Critical appraisal	
References	First author	Main research area and setting	Number	Participants	CASP score	CASP points lost/unclear
[21]	Charani E	Development of stewardship programmes in different countries. (SSA: Burkina Faso)	52	Healthcare professionals (doctors and pharmacists)	7/9	No. 5: No clear description of interview technique No. 6: Lacking discussion of relationship between researcher and participants
[28]	Legenza L	Healthcare provider knowledge about *Clostridium difficile* infection in a South African hospital	26	Healthcare professionals (doctors, nurses and pharmacists)	8/9	No. 6: Lacking discussion of relationship between researchers and participants
[27]	Gebretekle GB	Implementation of antibiotic stewardship in an Ethiopian tertiary care hospital	35	Healthcare professionals (doctors, pharmacists)	8/9	No. 4: Subjective criteria for recruitment, focussing on prestige of job role
[32]	Mula CT	Workarounds and their perceived impact on antibiotic stewardship in a referral hospital in Malawi	13	Healthcare professionals (nurses)	7/9	No. 4: No discussion of recruitment criteria for interview No. 6: Lacking discussion of relationship between researchers and participants
[31]	Rout J	The role of ICU nurses in antimicrobial stewardship at a South African private hospital	17	Healthcare professionals (doctors, nurses and pharmacists in ICU/surgery)	8/9	No. 6: Lacking discussion of relationship between researchers and participants
[19]	Torres NF	Patterns of self-medication in Maputo city, Mozambique	49	Healthcare professionals (pharmacists) and pharmacy customers	9/9	No points lost
[24]	Agardh C	Using pharmacists and drugstore workers as sexual healthcare workers among MSM in Dar es Salaam, Tanzania	15	Drugstore customers (men who have sex with men)	9/9	No points lost
[29]	Asante KP	Knowledge of antibiotic resistance and prescription practices in the Brong Ahafo Region of Ghana	33	Healthcare professionals (doctors, physician assistants, nurses and community health officers)	8/9	No. 8: Brief description of data analysis technique for qualitative component
[20]	Gebretekle GB	Exploration of over-the-counter sales in community pharmacies in Addis Ababa, Ethiopia	5	Healthcare professionals (pharmacists)	7/9	No. 4: Recruitment sequential, may not have achieved full geographical coverage No. 5: Interview technique not discussed
[22]	Yantzi R	Antibiotic use for viral respiratory infection in rural southwestern Uganda	22	Healthcare professionals (clinical officers, nursing offices, nurses, laboratory staff, non-medical staff, public health professionals, village health team members)	6/9	No. 4: Recruitment strategy for non-host clinic participants unclear No. 5: Interview technique not discussed No. 6: Lacking discussion of relationship between researchers and participants
[23]	Watkins JA	Community perceptions of antibiotic usage in Mpumalanga province, South Africa	17	Community members randomly selected from a Health Demographic Surveillance site	9/9	No points lost
[25]	Foster EK	Patient knowledge of prescription medications and antibiotics in Blantyre, Malawi	54	Pharmacy customers at 5 randomly selected community pharmacies	8/9	No. 5: Interview technique/questions asked not discussed
[30]	Pearson M	Awareness of anti-microbial resistance in multiple low-income countries (SSA: Ethiopia, Nigeria and Sierra Leone)	244	Healthcare and veterinary professionals (doctors, dentists, nurses, pharmacists, educator, veterinarian)	9/9	No points lost
[26]	Dillip A	Factors influencing antibiotic dispensing in Tanzania	84	Healthcare professionals (pharmacy owners and dispensers)	9/9	No points lost

Eight concepts emerged upon the first round of coding, which were condensed into 5 main themes: ineffective regulation, healthcare system factors, clinical governance, patient factors and lack of resources. The original concepts can be found in “Appendix 3”.

### Ineffective regulation

This theme describes a lack of regulation at country or region-level of resistance-promoting behaviours. Torres et al. noted that while there are laws against OTC sale of antibiotics in Mozambique these were rarely enforced [19]. Moreover, all pharmacy workers interviewed in Addis Ababa by Gebretekle et al. [20] mentioned that the weak or non-existent enforcement of regulation was a major driver of inappropriate dispensing.

There is an apparent tension between medicines access and regulation. This was highlighted by Charani et al. [21], stating that while tightening regulations would probably lower the rate of OTC antibiotic sales it could also reduce access to medications if drug sellers were shut down. This is supported by Yantzi et al. [22], who added that more remote communities, who could often not afford to travel to a clinic to obtain a prescription, would be disproportionately affected by this.

### Healthcare system factors

This theme relates to the nature of the healthcare systems in SSA encouraging resistance-promoting behaviours. It was sub-divided into *health system heterogeneity* and *pharmacies as a business*.

#### Health system heterogeneity

Healthcare professionals interviewed in Burkina Faso stated that patients often saw a combination of local healers, pharmacists, private and public healthcare services regularly [21]. This system allows patients to ‘shop around’ for a service that will provide antibiotics [21]. It also limits a clinician’s ability to obtain an accurate drug history, making it challenging to prescribe an antibiotic the patient has not recently received [21]. Complex healthcare systems are also harder to regulate, with some authors noting that this is further complicated by the black market and more targeted medication sellers such as ‘pension markets’, which are aimed at older adults [21, 23].

#### Pharmacies as a business

Interviews of drug store customers in Dar es Salaam indicated that if a pharmacy refused to sell antibiotics then customers would simply go to another [24]. Pharmacy workers interviewed by Gebretekle et al. [20] reinforced this, adding that pharmacy owners would reprimand or dismiss workers who refused sales on the grounds of stewardship. Equally, while many pharmacy customers in Blantyre felt that it was reasonable to be denied antibiotics unless they had a prescription, many also argued that pharmacies were primarily businesses and thus should never refuse sales [25]. This was also highlighted by Dillip et al. [26]. They found that even among Tanzanian accredited drug dispensing outlets, which are certified to follow national dispensing guidelines, inappropriate antibiotic dispensing was common due to the need for profit and the fear that customers would simply go elsewhere [26].

### Clinical governance

This theme relates to lack of AMS guidelines or lack of adherence to them. Gebretekle et al. [27] found that a major barrier to implementation of AMS programmes in an Ethiopian tertiary care hospital was a lack of support for AMS policy at institutional and national level. Furthermore many junior physicians routinely prescribed “safe” broad-spectrum antibiotics out of fear of receiving a negative career evaluation if they used a narrow-spectrum one [27]. This was echoed by physicians in surgical wards who would prolong the use of pre and post-operative antibiotics to prevent infectious complications which they would be blamed for [27].

In Legenza et al.’s [28] study in South Africa only 30% of clinicians knew about *C. difficile* guidelines, with even fewer being able to correctly recall them. Furthermore, many healthcare professionals interviewed in Ghana repeatedly prescribed antibiotics based on personal preference and experience rather than referring to guidelines [29]. This was also true of prescribers interviewed by Pearson and Chandler and Yantzi et al. [22, 30]. Additionally it was apparent that affordability and physical availability of antibiotics often dictated prescriptions more than guidelines [30]. Finally Yantzi et al. [22] added that prescribing a drug is often considered synonymous with a high standard of care by patients; adding to the pressure on clinicians to ignore stewardship guidelines. Adherence to guidelines was also examined by Rout and Brysiewicz [31], who argue that members of staff specifically trained to safeguard stewardship could help alleviate some of these problems.

Five papers in our study assessed the knowledge level of healthcare staff and they found that AMR is generally perceived as a significant threat, although this did not always translate into practice [21, 26, 27, 29, 30]. Pharmacy workers interviewed by Dillip et al. [26] in Tanzania could all correctly recite national antibiotic prescribing guidelines but all also admitted to ignoring these guidelines. Furthermore Gebretekle et al. [27] found that 90% of interviewed physicians recognised AMR as a national threat but more than half could not identify what organisms commonly caused resistant infections in their region.

### Patient factors

This theme refers to actions by patients which encouraged resistance-promoting behaviour by healthcare professionals such as inappropriate dispensing of antibiotics.

It was commonly reported that patients recognised and remembered certain drugs and the symptoms they were prescribed for. This allowed them to demand antibiotics from the pharmacist directly, rather than attend a clinic or hospital first which was perceived by patients as a waste of time and/or money. This was a dominant theme in Torres et al.’s [19] study in Mozambique. Many of the patients in this study knew the exact name and dose of drug they wanted [19]. Similar patterns were illustrated in all four of the included studies that interviewed patients [19, 23–25].

Another paradigm explored by Agardh et al. [24] was that marginalised communities, such as men who have sex with men (MSM), may prefer to only visit pharmacies. This is because pharmacies require less information about their personal lives and are in less public places, reducing the chance of encountering members of their community who may enquire why they are receiving medication [24].

### Lack of resources

This theme constituted a lack of the facilities required to appropriately prescribe antibiotics and overstretched health services necessitating practices that promote resistance. It was an over-arching theme that appeared in several of the other themes.

Four studies mentioned that a lack of laboratory facilities prevented antibiotic prescribing based on sensitivity testing [27–30]. Without sensitivity information clinicians must rely on resistance-fostering broad-spectrum antibiotics. Moreover, Legenza et al. [28] found that limited clinician time and a lack of IT infrastructure meant that often only the available cultures perceived as “more important” are checked.

The issue of limited ward time was echoed by Mula et al. [32] who studied ‘workarounds’: short-cuts taken on wards to reduce the time spent on certain tasks. Relevant examples include issuing rounded-up doses or simplified regimens that patients are more likely to understand and take less time to explain [32]. While these are arguably necessary due to the significant shortfall in healthcare staff, they also contribute to AMR.

The aforementioned problem of patients going straight to pharmacies also has roots in the lack of healthcare resources. Long wait-times at clinics, principally due to inadequate staffing, make skipping them an attractive option [20, 22]. Equally many healthcare facilities in SSA have a very limited range of available antibiotics, resulting in patients being prescribed the same antibiotic on every encounter [19]. This increases the likelihood of patients remembering the drug name and dose and, in combination with the internet, is a major driver of OTC sales according to Torres et al. [19].

## Discussion

### Key findings

To our knowledge this is the first systematic synthesis of qualitative studies surrounding antibiotic resistance in SSA. Studies were found for 11 out of the 46 SSA countries, and this lack of coverage is in keeping with findings from systematic reviews of surveillance data [4]. Additionally, many of the issues identified are either due to or exacerbated by the lack of resources in the study countries. Indeed, one could argue that healthcare system heterogeneity as a whole is a symptom of under-resourced healthcare.

Lack of resources is not solely responsible for AMR in SSA and several contextual factors were repeated throughout the included studies. There was consensus that a tension existed between a pharmacy worker’s role in upholding antimicrobial stewardship and the need for profit in a highly competitive economy. There was also conflict between the need for regulation of drug stores and the risk of limiting access to medications. Several studies highlighted the fact that many patients see going to a clinic as expensive and time-consuming when they can simply demand OTC sale direct from the dispensary. It is also apparent that despite ongoing efforts to educate staff about antimicrobial stewardship, resistance-promoting behaviours still occur in clinics and hospitals for a variety of reasons.

### Comparison with existing literature

Our findings have much in common with a 2016 review of the implementation challenges of global antimicrobial stewardship by Tiong et al. [33]. They argue that while there is a lack of resources, many stewardship interventions themselves are categorically at odds with developing economies [33]. Specifically, they cite the balance between regulation and access to medications as an example of this disconnect between policy and practice [33]. We also agree with Van Dijick et al. [34], who state that the literature base surrounding stewardship interventions is heterogenous and complicated by a myriad of sociocultural paradigms unique to each country within SSA. Furthermore our main themes bear considerable resemblance to the findings of Kpokiri et al. and Huttner et al., who published studies analysing the implementation of antimicrobial stewardship programmes in Nigeria and across the globe, respectively [35, 36]. In particular, we share their sentiment that further publication of evaluation of stewardship interventions, regardless of their success, is exceedingly valuable to inform future efforts.

When comparing literature it should be noted that the health systems of SSA are far from identical. One example is that in one study in Ethiopia all interviewed pharmacy workers either held a bachelor’s degree in pharmacy (B. Pharm) or a diploma in pharmacy [20]. Meanwhile in Tanzania the level of qualification of pharmacy worker depends on the type of pharmacy and can range from a degree-level pharmacist to any individual with a medical background, such as a nurse [24]. Furthermore, the development and enforcement of antibiotic prescribing guidelines varies greatly between different countries in SSA, and few countries have a national AMR policy [7, 37]. These differences reinforce the need to tailor stewardship interventions to individual countries.

Patient education, while out of the scope of this review, should also be considered when evaluating our findings. Torres et al. conducted a systematic scoping review of factors influencing self-medication with antibiotics in low and middle income countries [38]. They found that patients who possessed low or very high knowledge of antibiotics were the most likely to engage in self-medication [38]. Some of the papers in our study also discussed this, with Gebretekle et al. finding that those with specific knowledge on antibiotics were better equipped to specifically request them while Watkins et al. reported that very few patient interviewees knew of AMR as an issue [20, 23]. While it has a small literature base, patient education impacts resistance-promoting behaviours and thus should be included both in future interventions and research efforts.

Intertwined with patient education on AMR is general health literacy [39–41]. This also varies among SSA countries, though there is little data in this field [39–41]. One study of 224,751 individuals from 14 SSA countries found an average prevalence of high health literacy of 37.55% and a range of 8.93% (Niger) to 63.89% (Namibia) [39]. A systematic review by Castro-Sánchez et al. [41] suggests that there is a relationship between health literacy and antibiotic usage, but it is complex and as yet not fully understood. Furthermore, this relationship does not appear to have been studied in SSA outside of South Africa [41]. Health literacy is therefore likely another important contextual factor in stewardship in need of further research.

While the included literature showed reasonable consensus on the levels of knowledge of AMR among staff, this is not replicated in wider literature. Labi et al. found that 8.9% (14/157) physicians in a Ghanaian tertiary care hospital considered AMR a threat locally, while Erku reports that 26.5% of 449 community pharmacists interviewed in Ethiopia believed that stewardship should be practised by drug stores [42, 43]. Studies from both Ghana and Ethiopia in this review found that more than half of the healthcare staff interviewed at least acknowledged AMR as a threat [27, 29]. This reinforces the fact that this literature base is far from complete and more data is required.

### Limitations

This study has a number of limitations. The literature search was conducted in English meaning that manuscripts in other languages could have been missed. While translated papers were found and included in the abstract screening, none met the inclusion criteria. Equally, the lack of geographical coverage constitutes a reporting bias, as countries where AMR is considered a less important issue are less likely to commission research into it. We did not include articles specifically on veterinary practice, another important source of AMR [44]. Finally, there is the potential for publication bias. African nationals underrepresented in academia, and may find it more difficult to publish papers in major journals due to a lack of resources or a lack of interest on the part of the journals [45].

## Conclusion

Antibiotic resistance is a growing problem and could significantly undermine healthcare in SSA. Lack of data is a major barrier to any public health interventions in this field. Therefore, wider surveillance and reporting of resistant infections along with further research into its underlying drivers are needed. Specifically, research in countries which are not currently included in the literature base should be prioritised. Moreover, funding, publication and evaluation of stewardship interventions, successful or not, could help inform future endeavours and inspire action among policy-makers. It is important to recognise that stewardship and resistance do not exist in isolation and are part of wider healthcare systems. Increased regulation seems an obvious course of action but must be balanced with continuing access to medications. Financial incentives to drug stores that comply with regulation, rather than closing those than do not, could be an acceptable middle ground in this regard. Equally, increased national and regional support for stewardship could improve its priority in a clinical setting. In summary, while increased health resources will help AMS efforts in SSA, specific interventions tailored to the unique context of the region, are also required.

## Data Availability

All papers included in this review are available in the ‘References’ section. A full search strategy, along with the original thematic analysis spreadsheet, is available from the corresponding author on request.

## Appendix 1: Search strategy for embase

**Table:**

Search term	Number of results
Qualitative research/	71,954
Antimicrobial stewardship.mp. or exp antimicrobial stewardship/	6749
Exp “Africa south of the Sahara”/	223,132
1 and 2 and 3	8

**Table:**

Search term	Number of results
Antibiotic resistance.mp. or exp antibiotic resistance/	162,413
Exp “Africa south of the Sahara”/	223,123
Qualitative research.mp. or exp qualitative research/	82,173
1 and 2 and 3	11

**Table:**

Search term	Number of results
Exp antimicrobial stewardship/ or exp antibiotic resistance/	154,784
Exp “Africa south of the Sahara”/	223,435
Survey.mp	1,095,152
Cross-sectional study.mp. or exp cross-sectional study/	373,722
1 and 2 and 3 and 4	42

**Table:**

Search term	Number of results
Cross-sectional survey.mp. or exp cross-sectional studies/	328,594
Exp “Africa South of the Sahara”/	203,956
Exp antimicrobial stewardship/	1183
1 and 2 and 3	2

**Table:**

Search term	Number of results
Exp “Africa South of the Sahara”	203,956
Antibiotic resistance.mp. or exp drug resistance, microbial/	176,519
Survey.mp. or exp “Surveys and Questionnaires”/	1,320,095
1 and 2 and 3 and 4	93

## Appendix 2: Search Strategy for Ovid Medline(R) and in-process and other non-indexed citations

**Table:**

Search term	Number of results
Antibiotic resistance.mp. or exp drug resistance, microbial/	176,214
Qualitative research.mp. or exp qualitative research/	63,788
Exp “Africa South of the Sahara”/	203,519
1 and 2 and 3	11

**Table:**

Search term	Number of results
Antibiotic stewardship.mp. or exp antimicrobial stewardship/	2703
Qualitative research.mp. or exp qualitative research/	63,826
Exp “Africa South of the Sahara”/	203,519
1 and 2 and 3	3

## Appendix 3: Original concepts

These were the original thematic concepts identified by GJP and then validated and consolidated into themes by GJP and the co-authors in light of the fully coded papers.

**Table:**

Theme	Explanation	Papers expressing theme
Ineffective regulations	This encompassed comments from several papers about regulations either being absent or poorly enforced. This allowed patients to procure prescription-only medication such as antibiotics without a prescription. Interestingly, several studies argued that rigid enforcement of regulations would cut off the supply of medications to certain communities who found it difficult to get a prescription in the first place	[19–22]
Heterogeneity in healthcare	Many of the study countries had very heterogenous healthcare systems, with patients seeing traditional practitioners, private clinics and government healthcare. This promoted patients passing through multiple different healthcare branches, taking many medications and not having accurate medical records available	[21]
Black market supply	Black market suppliers were reported specifically in 2 papers. These are hard to regulate and increase illicit access to prescription-only medications	[21, 23]
Lack of resistance reporting infrastructure	This was reported in five papers. This encompassed a lack of the culture machines and/or laboratories necessary for the timely reporting of sensitivity results. This made it difficult for clinicians to prescribe appropriate narrow-spectrum antibiotics, relying instead on broad-spectrum ones which are more likely to foster resistance	[27–30]
Bigger clinical concerns	This was reported in three papers. In many cases, issues such as tuberculosis, HIV and tropical diseases formed a much greater concern on wards. This meant that little attention was paid to resistant organisms	[27, 28]
Clinical governance	This was reported in many papers, and encompassed a lack of appropriate stewardship guidelines (or a lack of adherence to them) at the level of individual wards and pharmacies. There were many reasons for this, such as lack of knowledge by clinical staff or fear that prescribing a narrow-spectrum antibiotic would result in a negative career validation. It also involved pharmacies needing to make a profit, and doing so by selling prescription-only medication to patients without appropriate prescriptions	[19, 20, 22, 24–31]
Lack of equipment	This encompassed a lack of resources at the level of wards and departments. In some cases, this resulted in only having access to a small number of antibiotics, causing patients to learn their names and increasing the ease of which they were procured. In other cases it was a lack of ward time to devote to checking culture results and altering prescriptions	[22, 28, 31, 32]
Patient factors	Another wide-reaching theme, this was specific patient behaviours that fostered antimicrobial resistance or hampered efforts for antimicrobial stewardship. Some examples included patients ‘shopping around’ for a healthcare service that would sell them antibiotics regardless of prescription, self-medication, or when patients would learn the dose and name of an antibiotic so they could directly request it from a pharmacist without a prescription	[19, 20, 22–26, 29]

## Abbreviations

AMR: Antimicrobial resistance
CASP: Critical Appraisal Skills Programme
OTC: Over the counter
PRISMA: Preferred Reporting Items for Systematic Reviews and Meta Analyses
SSA: Sub-Saharan Africa
WHO: World Health Organization
